# Treatment and Monitoring of Eosinophilic Fasciitis

**DOI:** 10.1007/s40674-024-00222-6

**Published:** 2025-01-23

**Authors:** Albert Selva-O’Callaghan, Ernesto Trallero-Araguás, Albert Gil-Vila, Alfredo Guillen-Del Castillo, Ana Matas-García, Jose Cesar Milisenda, Carmen Pilar Simeon-Aznar, Iago Pinal-Fernandez

**Affiliations:** 1 Systemic Autoimmune Diseases Unit, Internal Medicine Department, Vall d’Hebron General Hospital, Universitat Autònoma de Barcelona, Barcelona, Spain; 2 Rheumatology Department, Vall d’Hebron Hospital, Barcelona, Spain; 3 Muscle Research Unit, Internal Medicine Service, Hospital Clinic, Barcelona University, CIBERER and IDIBAPS, Barcelona, Spain; 4 Muscle Disease Unit, National Institute of Arthritis and Musculoskeletal and Skin Diseases, National Institutes of Health, Bethesda, MD, USA; 5 Department of Neurology, Johns Hopkins University School of Medicine, Baltimore, MD, USA

**Keywords:** Eosinophilic fasciitis, Immunosuppressive treatment, Monitoring, Unmet needs, Groove sign

## Abstract

**Purpose of Review:**

Eosinophilic fasciitis (EF) is a rare inflammatory disease characterized by skin induration. Although some guidelines from scientific societies exist, standard recommendations on monitoring and therapy are lacking.

**Recent Findings:**

Current therapy for patients diagnosed with EF includes glucocorticoids plus at least one immunosuppressive drug in cases of relapse or refractory disease. Methotrexate and mycophenolate mofetil are the most recommended, although recently a myriad of case reports or small series reporting the effectivity of biological agents or JAK inhibitors for treating relapses or refractory disease have been published. Anti-IL5 may have a role in those rare refractory cases with persistent eosinophilia. Intravenous immunoglobulins and photopheresis (in those centers with experience) may act as adjuvant therapies. Monitoring the disease activity is a cornerstone to ascertain if the treatment is useful or not. MRI, PET/TC, and more specifically POCUS have recently demonstrated their value for assessing therapy response.

**Summary:**

High-quality data focused on therapy and monitoring is lacking in EF. Strategies for improving scientific quality of observational studies and consensus about “activity”, “sequela”, “relapse” or “refractoriness” terms in EF patients are necessary to implement prospective clinical trials and generate evidence-based medicine. Meanwhile we have to deal with the available information.

## Introduction

**Fig. 1:**
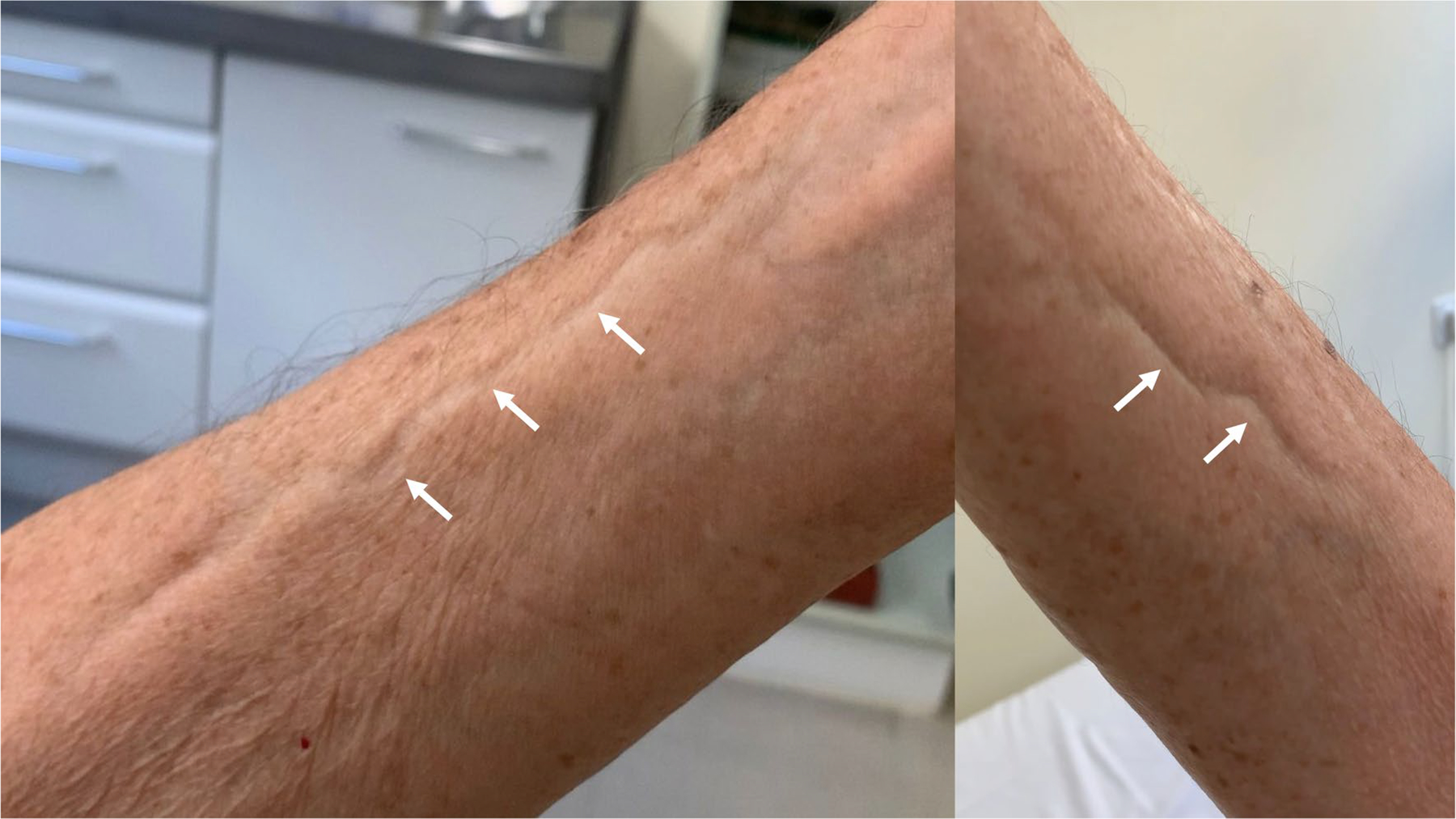
Groove sign (forearm, arrows)

**Fig. 2:**
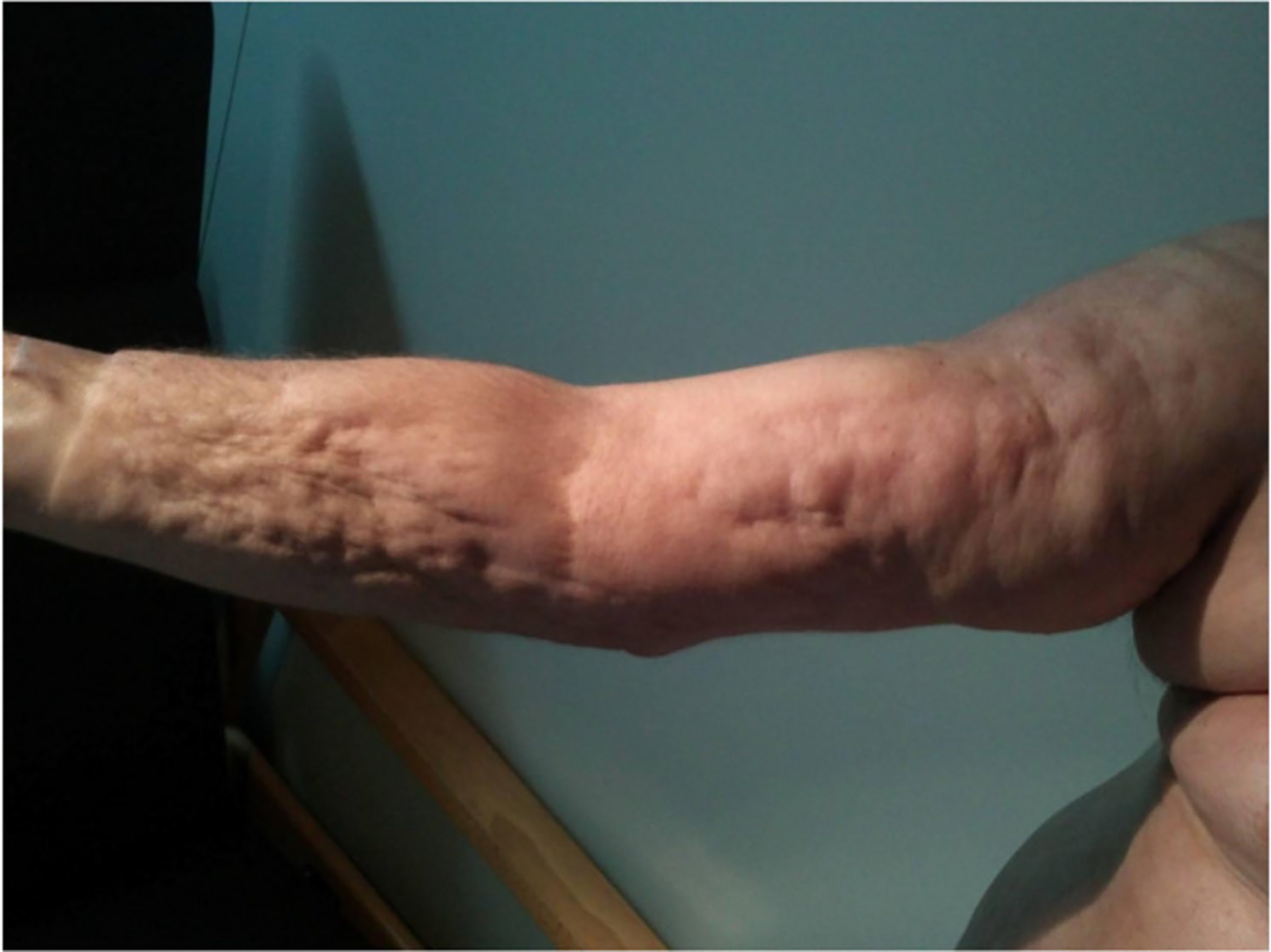
Peau d’orange

Eosinophilic fasciitis (EF) was first described by Shulman in 1974 [1], and after the first large cohort was published in 1988 [2], it was accepted by the medical community as a new entity. Eosinophilic fasciitis is characterized by local or diffuse skin induration, with the characteristic “Groove sign” (Fig. 1, arrows), a depression along the course of the superficial veins, more marked on the elevation of the affected limb [3, 4]. The onset of the disease is usually abrupt, with painful swelling of the affected limbs symmetrically or not, it may involve also the trunk or abdomen, which is progressively replaced by skin induration and several weeks later the classical “*peau d’orange*” appears (Fig. 2) [5, 6]. Eosinophilic fasciitis is an inflammatory disease of unknown etiology occasionally linked to a previous vigorous exercise [7–10]. Hematological disorders including malignancies, myelodysplastic syndromes, or monoclonal gammopathies have also been described as associated with EF in the reported series [2, 7, 11]. Checkpoint inhibitor therapy, which includes PD-1and PD-1L blockers, is widely used in several types of cancer, primarily melanoma and lung adenocarcinoma. Eosinophilic fasciitis has recently been reported as one of the possible immune-related adverse events of this immunotherapy [12, 13].

Peripheral eosinophilia, mainly at disease onset is a diagnostic “red flag” for the astute clinician in patients with skin induration, although it disappears quickly after the administration of prednisone therapy. Hypergammaglobulinèmia and mildly elevated aldolase levels are also characteristics of the disease [5, 6]. A sequential apparition of the different skin manifestations is observed in these patients: edema with Groove sign at onset, fibrosis with morphea and *peau d’orange* afterwards, and disability or joint contractures as late sequela.

Eosinophilic fasciitis can be considered a scleroderma-like disorder. However, from a clinical point of view it is not difficult to differentiate between both entities. The absence of Raynaud’s phenomenon, skin induration that respects the acral part of extremities, and, in general, sparing of internal organs are some of the hallmarks of EF. Moreover, antinuclear antibodies are negative, and no specific autoantibodies are linked to the disease, on the contrary, the characteristic anti-topoisomerase I (anti-Scl70), anti-centromeric, or anti-RNA polymerase III, which may be positive in systemic sclerosis, are herein negative.

Lymphocyte infiltration and thickening of the fascia are the typical signs observed after a full-thickness skin biopsy of the affected area [5]. The presence of an eosinophilic infiltrate into the fascia is not mandatory for the diagnosis.

Current treatment includes the administration of glucocorticoids as a mainstay therapy. In some patients, the relapse of the disease or the chronic outcome prompts to the concomitant administration of immunosuppressive drugs such as methotrexate, mycophenolate mofetil, or intravenous immunoglobulins.

## Treatment

### Diet and lifestyle

Not specific diet recommendations are issued to protect the development of EF. However, it is known that several diet supplements for example L-tryptophan in the case of the eosinophilia-myalgia syndrome [14, 15], or toxic substances such as the toxic oil that was involved in the toxic oil syndrome [16] have been linked to EF.

Strenuous physical exercise has been described in several series as a triggering factor of the disease [7–10], but not in others [6, 17, 18]. However, physicians should be cautious to recommend against exercise in these patients or in healthy patients to avoid the development of this rare disorder, given the well-known benefits ascribed to usual physical exercise. A cautious risk–benefit ratio evaluation is needed in these patients.

Those patients diagnosed with EF are recommended for the usual schedule of vaccination that is endorsed for the general population, that means for influenza, pneumococcal, *Haemophilus influenzae*, hepatitis B virus, and SARS-CoV-2. Recombinant varicella-zoster vaccine is also strongly recommended in those patients receiving some specific therapies such as JAK inhibitors (i.e., ruxolitinib, tofacitinib) if it is the case in refractory disease [19, 20]. Given that some cases of EF have been described in patients after influenza or SARS-Cov2 vaccination [21, 22], this issue should be deeply discussed with the patient and again bearing in mind the individual risk–benefit ratio.

### Pharmacologic treatment

The first step before evaluating the effectiveness and utility of the different pharmacological approaches in patients with EF is to define what we refer to as a “treatment response”. No consensus exists for such an issue, although in some studies the authors made an important effort to establish this definition [23].

Patients with persistent active physical signs and symptoms, at the criteria of the physician in charge, are defined as having “failure to therapy”. Those with a remaining disability, for example, persistent joint contractures, tendon retraction or subcutis sclerosis, without active signs or symptoms attributable to EF will be defined as “remission”; and only we refer to “complete remission” when the patient is free of symptoms at the end of follow-up with resolution of physical findings. The presence of disability or failure of therapy defines a poor outcome. Eosinophil count is relevant for the diagnosis but not a good marker of activity, given that administration of corticosteroids reverts the hypereosinophila very fast.

#### Glucocorticoids

It is widely accepted and is our experience, that corticosteroid therapy is the mainstay therapy for treating EF [17, 18, 23]. In several published series, this is the first step (first-line therapy) of treatment and more than half of patients achieve a partial or complete remission [2]. Standard doses of prednisone are usually administered (1 mg/kg/d) during the first month after diagnosis with progressive reduction over 3–6 months. Physicians sometimes recommend low-dose corticosteroids (2.5 mg/d prednisone) as a maintenance therapy. *Pneumocystis jirovecci* prophylaxis and antiresorptive therapy for avoiding undesirable side effects are recommended on a case-based strategy. Although the benefit of methylprednisolone pulses administration (500–1,000 mg/d × 3 consecutive days) is not evidence-based supported, in a study of 34 patients diagnosed with EF published in 2012, the authors found that those who did not receive methylprednisolone pulses had a poor outcome than the patients who received such therapy [17].

#### Methotrexate

(7.5–25 mg/week) [24] is the drug usually recommended as a second-line therapy when corticosteroids alone are not effective for achieving complete remission, when the disease relapses, or due to unacceptable adverse events such as osteoporotic fractures, hyperglycemia or stigmas of hypercortisolism. Also, in some clinical situations such as patients who develop some morphea-like lesions, this drug should be considered as a second-line therapy together with the administration of corticosteroids. Other immunosuppressive drugs such as azathioprine (1–2 mg/kg/day), cyclosporin (100–150 mg/day), biologic agents (rituximab, tumor necrosis factor blockers, interleukin-6 receptor inhibitors) and hydroxychloroquine are also included as a second-line therapy in EF [7, 8, 17, 18, 23]. Mycophenolate mofetil (1 g/12 h) plus intravenous immunoglobulins (0.4 gr/kg/d × 5 days, once a month × 6 months) may be useful in severe situations such as perymyositis or restrictive respiratory failure due to trunk skin induration [25–28].

Other series [18] such as Chaigne et al., supported the role of increasing corticosteroids with the addition of methotrexate as a strategy for treating relapses of the disease that may be linked to the presence of fibrosis. In the same study the authors establish through an unsupervised cluster analysis 3 different clusters (“mild”, “late-onset and hypereosinophilia”, and “fibrosis”) that may be potentially useful for stratifying the therapy. However, it is not clear that these phenotypes may be able to separate and stratify the treatment response.

#### Mycophenolate mofetil

This drug is one of the glucocorticoid-sparing agents used for long-term treatment in patients with EF [26, 28]. In a retrospective study from 3 tertiary centers published in 2020, the authors analyzed 14 patients diagnosed with eosinophilic fasciitis who had been treated with mycophenolate mofetil (maximum daily dose between 2,000 and 3,000 mg) as a glucocorticoid-sparing agent. At 6 months after the diagnosis 10 patients (71%) had a partial response (halted disease progression with incomplete improvement of erythema and edema or improvement in all parameters other than induration) and only 3 (21%) had a complete remission (halted disease progression, resolution of erythema and edema, and improvement of induration) that increased to 7 patients (50%) after 1 year of treatment.

One hundred percent (12 out of 12) of patients with baseline functional impairment, defined as joint contractures or restricted mobility. treated with mycophenolate mofetil improved after 1 year of treatment [26]. Minor gastrointestinal adverse events (nausea, dyspepsia, vomiting…) appeared in 7 patients (50%) but in most cases resolution of the symptoms was achieved over time or after switching to mycophenolic acid.

A recent literature review analyzed 27 cases of patients diagnosed with eosinophilic fasciitis who received mycophenolate mofetil or mycophenolic acid as a sparing-glucocorticoids agent. A total of 19 patients had a complete response, and 6 had a partial response. Only 2 cases were refractory to the combination of glucocorticoids plus mycophenolate [28].

Thus, mycophenolate mofetil is a good option as a second-line therapy for patients with EF. The rationale of its efficacy seems to rely on the inhibition of the TGF-β pathway and the consequent decrease of fibroblastic activity and collagen synthesis [29]. This antifibrotic effect in addition to the immunomodulatory properties, supports this drug as a first-line glucocorticoid-sparing agent.

### Biological agents

#### Tocilizumab

IL-6 blocking by a humanized monoclonal antibody, tocilizumab, recently has gained interest in the treatment of EF when other more conventional agents such as methotrexate or mycophenolate mofetil are not working as a second-line glucocorticoid-sparing agents [30–34]. Collagen stimulation and participation in the physiopathology of skin fibrosis are some of the well-known roles of IL-6 as has been postulated in other fibrosing disorders such as systemic sclerosis, although clinical trials addressed to this point did not achieve significant results [35, 36].

#### Rituximab

A chimeric monoclonal antibody directed against CD20 antigen in B-cells (anti-CD20) has demonstrated its utility in the treatment of several autoimmune diseases such as rheumatoid arthritis [37], ANCA-associated vasculitis [38], or systemic sclerosis [39]. A total of 8 cases of patients with refractory EF have been reported who successfully respond to rituximab. Most of the cases reported received therapy with 2 to 4 immunosuppressive drugs including azathioprine, methotrexate, hydroxychloroquine, cyclophosphamide, mycophenolate, etanercept, golimumab or cyclosporin, before rituximab therapy. Only 2 cases did not respond to rituximab. Unfortunately, there is no recommendation regarding the dose or duration of the treatment, however, common sense suggests that the dose should follow the usually administered in most of autoimmune disorders (1 g given twice within a 2-week interval). Thus, this biological therapy could be a therapeutical option in severe and refractory cases of EF [40, 41].

#### Infliximab

A chimeric monoclonal antibody against the TNF-α has occasionally been used in the therapy of patients with EF refractory to conventional treatment. TNF is a proinflammatory cytokine and the use of monoclonal antibodies directed against this molecule was a successful treatment in systemic diseases such as Crohn`s disease or specifically rheumatoid arthritis or psoriatic arthritis. Khanna et al. [42] reported three cases of refractory EF that improved after the administration of infliximab 8 weeks after starting this therapy and a drug-free remission 1–3 years after the onset of treatment. Even the joint contractures, classically attributed to sequela, improved in these patients. Two more cases previous to the report of Khanna et al. were published and in both of them there was an improvement of the main symptoms [43, 44].

#### Intravenous immunoglobulins

Therapy with IVIGs has demonstrated interest and effectiveness in some systemic diseases, such as dermatomyositis, lupus or vasculitis [45–47]. Its use in patients diagnosed with EF may be directed to those patients with refractory disease and mostly in addition to other immunosuppressive drugs. Several case reports and our own experience seem to support their use in this clinical scenario [25, 26, 48]. In a large study performed by Tkachenko et al. [49], from 3 tertiary care centers the authors identified 5 patients with refractory EF, all being treated with prednisone and methotrexate, and in one case with the addition of mycophenolate mofetil. All patients were able to be withdrawn from glucocorticoids.

#### Anti-interleukine-5 (anti-IL-5) therapies

The rationale of this type of therapy relies on the theoretically pathogenic action of the eosinophils. It is well-known that IL-5 plays a role in the maturation, recruitment, and proliferation of eosinophils, thus it seems logical that monoclonal antibodies directed to the cytokine (mepolizumab or reslizumab, human monoclonal antibodies against IL-5) or to their receptor (benralizumab) may be useful in some cases that do not respond to the usual therapy with prednisone or immunosuppressive drugs. At least in those patients with sustained peripheral eosinophilia [50–53].

#### Janus kinase inhibitors

Patients with EF refractory to conventional therapy have been reported with a successful response to any one of the JAK inhibitors, baricitinib or tofacitinib [20, 21, 54]. The mechanism of action of these drugs is through blocking the signal transduction of several cytokines (IFN-α, IFN-γ, IL-2, IL-5, IL-10) that have been involved in the etiopathogenesis of the EF.

#### Bone marrow transplantation

Only two EF cases have been reported to be treated with bone marrow transplantation, one of them died early due to disseminated viral infection. Thus, the scarce evidence for using this therapy in clinical practice has to take with caution [55, 56].

#### Other drugs

Colchicine and D-penicillamine, have been advocated in the past as useful drugs for the treatment of patients with EF, but are not currently used in clinical practice [57]. Anecdotal reports suggest that sirolimus, a mechanistic target of rapamycin (mTOR) kinase inhibitor, that acts as a fibrosis regulator may be useful in refractory cases [58].

Guidelines from the Japanese Dermatological Association published in 2017 [23] establish treatment recommendations (see recommendation grades and evidence level in Table 1) about glucocorticoids effectivity for treating patients with EF (oral corticosteroid 1D, steroid pulse therapy, 1C); stop treatment after remission (2D); inefficacy of topical therapy (2D); and immunosuppressants as second-line treatment (2D). Primary Care Dermatology [59] Society is generally consistent with the Japanese Dermatological Association. A summary of the drugs, doses, and adverse events is reported in Table 2.

### Physical therapy

Extracorporeal photochemotherapy (ECP) or photopheresis, is based on the effects of UV radiation on the plasma enriched in lymphocytes collected from the patient by apheresis which is afterwards reinstituted. Although not well understood, the mechanism involved may be related to the massive release of cytokines after photopheresis and its modulatory effect on the immune response. Romano et al. [60] reported in 2003 their experience with 3 cases of EF, who did not respond to prednisone and cyclosporine. Again, the role of this technique is more as a coadjuvant therapy, being recommended in addition to an immunosuppressive schedule. After 1 year of therapy (two consecutive days at 2-week intervals for the first 3 months and thereafter every 4 weeks based on clinical response) 2 cases greatly improved and the other only slightly. In a recent review, Partarrieu-Mejías et al. [61], report a single case of refractory EF treated with photopheresis and review the current literature gathering 6 cases reported until now with a good response to this therapy.

UVA-1 phototherapy or irradiation (90 J/cm, for 40 consecutive sessions, 3–4 times a week) is an alternative therapy for difficult cases of EF. Data from 8 patients with EF after treatment with conventional immunosuppressive drugs is reported. It is not clear if this technique is useful or not [62]. Other forms of UVA-1 phototherapy have been reported, such as for example psoralen-ultraviolet bath photochemotherapy [63], or UVA-1 whole-body phototherapy plus psoralen [64]. The role of these therapies is difficult to ascertain, although if feasible in some centers probably is warranted and worth it.

### Surgery

Surgery approach to EF is uncommon. Only scarce studies, mainly case reports, have been published on this issue, related to forearm and leg compartment syndrome [65–67]. More frequent seems to be the case of carpal tunnel syndrome and median nerve compression, even as a first manifestation of the disease, probably due to tenosynovitis. Surgical decompression may be necessary in these cases. Thus, clinicians must be aware of these manifestations because a surgical approach may be necessary in some patients.

## Monitoring eosinofilic fasciitis

### Biomarkers

Serum biomarkers have been reported in patients with EF, although their specific role in monitoring the activity of the disease has not been elucidated. Serum levels of tissue inhibitor of metalloproteinase-1 and 2 which have a role in the regulation of extracellular matrix are higher in EF patients than in healthy controls but there is no data about the levels through the course of the disease [68]. The expression of soluble CD40 ligand suggests activation of lymphocyte T cells and has also been found at higher concentrations in the serum of patients with EF, with a good correlation with disease activity. The levels of CD40 ligand normalized and returned to normal values after treatment [69]. Other studies found a high value of serum levels of manganese superoxide dismutase -a protector of oxygen free radicals- in patients with EF in comparison with healthy controls, but there is not longitudinal data or follow-up [70]. Most of these studies, however, come from the same group of research and new data or confirmation by other groups have not been, to our knowledge, published in the literature, thus its value in clinical practice is difficult to ascertain.

On the other hand, more classical biomarkers associated with EF, besides the acute phase reactants (erythrocyte sedimentation rate and C reactive protein) include eosinophilia-although usually transient-, increased values of aldolase with normal CK levels, and hypergammaglobulinemia. Specifically, aldolase seems to be a good biomarker of activity as has been reported in several studies [71–73]. Some authors suggested that inflammation in superficial muscle fibers, near to the perimysium, a kind of fascia, releases aldolase more than CK [74].

### Image techniques

#### Magnetic resonance imaging

Magnetic Resonance Imaging (MRI) has been revealed as a useful non-invasive technique not only for the diagnosis of EF, but also to point at the more useful site for performing a full-thickness skin biopsy that will confirm the diagnosis. Characteristic findings include thickening of the fascia and contrast enhancement, usually sparing the muscle. T1-weighted with gadolinium contrast, fat-suppressed T2-wighted and short tau inversion recovery (STIR) are the most useful sequences [75]. Studies performed on a small series of patients and case reports demonstrate that MRI is also extremely useful for monitoring the activity of the disease [71, 76]. In a study reported by Bauman, the authors found that MRI findings disappear after the immunosuppressive therapy in a series of six patients diagnosed with EF [77]. A systematic review of medical literature recently published including 1703 patients diagnosed with EF, states that 76% underwent MRI at diagnosis although data focused on the utility of MRI for monitoring the disease was not reported [78].

#### ^18^F-FDG Positron emission tomography with computed tomography (PET/CT)

Whole-body non-contrast-enhanced ^18^F-FDG PET/CT is a non-invasive, hybrid technique has proven to be useful for detecting morphological and functional tissue changes in inflammatory disorders, and also as a cancer screening approach [79]. Recently case reports including PET/TC for diagnosis of EF have been published [76, 80, 81] suggesting that it could be a useful test for diagnosis and guiding the biopsy site. Moreover, in those patients diagnosed with EF and cancer, that represents a 10% of the cases, may help to diagnose the underlying malignancy.

#### Ultrasounds

This low-cost non-invasive tool, that may be performed at bedside is a promising and useful techniques. Recent exploit of POCUS (“point of care ultrasound”) in medicine will undoubtedly contribute to the dissemination of this diagnostic and monitoring practice [82, 83]. In patients with EF ultrasounds (US) may identify the thickened fascia with increased echogenicity that may be repeated as many times as needed, and thus is a good option for monitoring the disease activity [84–86]. A drawback of the technique is that it depends on the experience of physician in charge who is performing US, and that until now, to the knowledge of the authors, there is not a standardized score for evaluation.

### Unmet needs in eosinophilic fasciitis treatment and monitoring

There is a lack of high-quality information about which has to be the better treatment strategy in EF. Most of the data come from observational studies of retrospective nature, which include case reports and series of patients. Moreover, strategies and scores usually recommended for improving the scientific quality of these studies (i.e. STROBE) [87] are usually missing.

**Fig. 3:**
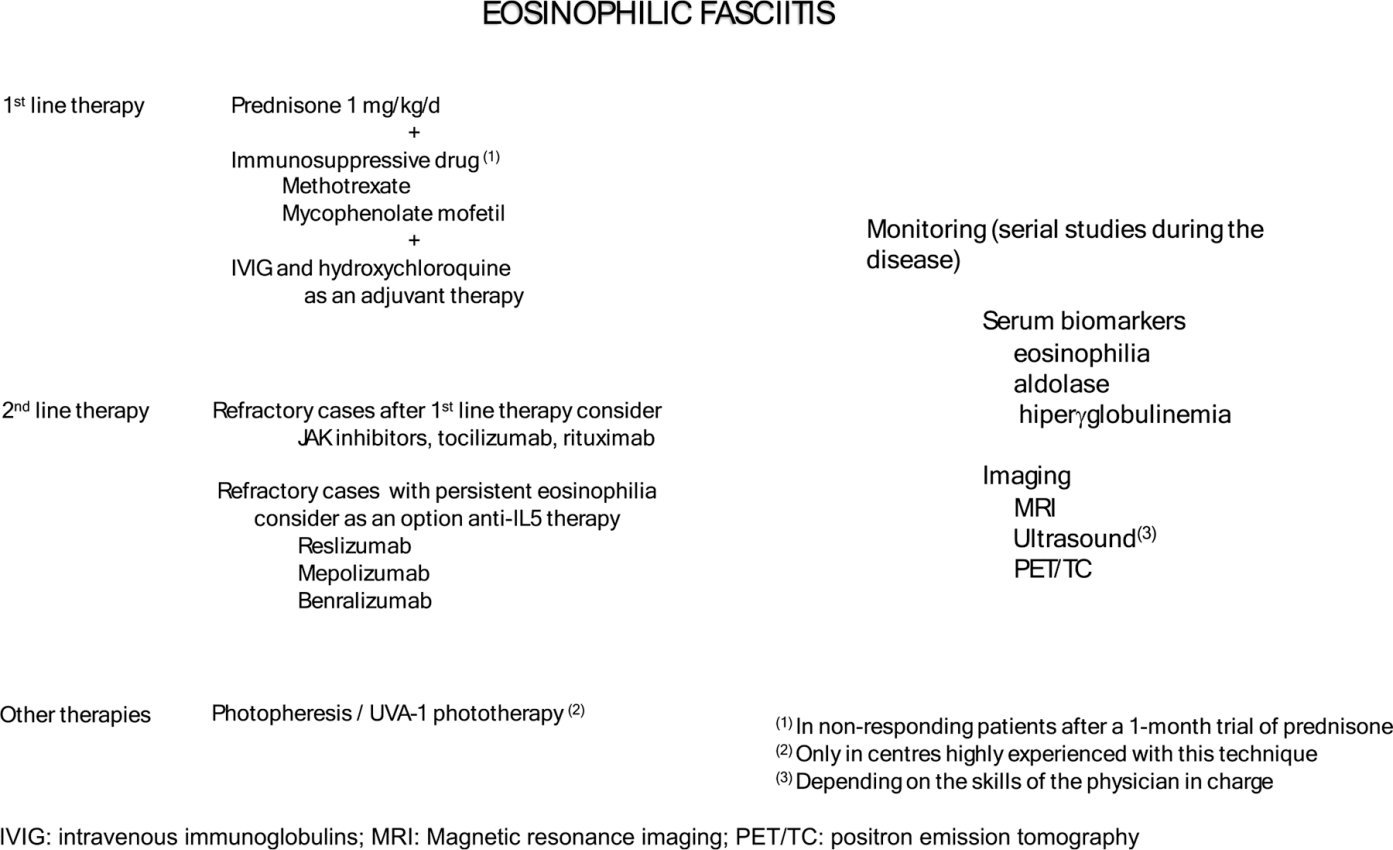
Management algorithm and monitoring in eosinophilic fasciitis

The following steps are needed to improve our approach to the therapy eosinophilic fasciitis in clinical practice, that may be gathered in a so-called management algorithm (Fig. 3). First, we need a consensus from the scientific community about the disease activity, sequela, or disability and also on the meaning of relapse and refractory disease. For succeed in this issue we need to integrate the known clinical signs and symptoms (induration, edema, fibrosis, Groove sign…), laboratory parameters, (aldolase, eosinophilia…) and image techniques (MRI, PET/CT…) or generate new ones that allow a better approach to these patients. The second, step, after achieving the first one is to generate evidence-based information with prospective clinical trials with different therapeutic approaches and drugs, and finally, the last step is to collect rationally all this data in a management algorithm that undoubtedly will be useful for clinicians and of course for the patients well-being.

## Figures and Tables

**Table 1:** Recommendations and evidence level classification used in Japanese Guidelines on eosinophilic fasciitis [23]

Recommendation grade	Evidence level classification
**1** Strongly recommended	**A** Evidence from systematic review/randomized controlled trial/ meta-analysis *OR* Evidence from at least one randomized controlled trial
**2** Advocated None when undecided	**B** Evidence from at least one controlled study without randomization
	**C** Evidence from analytical epidemiological studies (cohort study) *OR* Evidence from analytical epidemiological studies (case–control study, cross-sectional study)
	**D** Evidence from descriptive studies (case reports, case series) *OR* Evidence from expert committee reports or opinions or clinical experience of respected authorities, not based on patient data

**Table 2:** Second-line agents for the treatment of patients diagnosed with eosinophilic fasciitis

Drugs	Doses	Indications and comments	Side-effects
Methotrexate	7.5–25 mg/w s.c. or p.o	Relapse, morphea, refractory disease, corticosteroid tapering	Stomatitis, gastrointestinal symptoms, leucopenia, liver toxicity, infections, lung toxicity
Azathioprine	1–2 mg/kg/d p.o	Determine the level of TPMT	Gastrointestinal symptoms, myelosuppression, pancreatitis, liver toxicity
Hydroxychloroquine	200 mg/d p.o	Ophthalmologic control yearly	Gastrointestinal symptoms
Cyclosporine	150–300 mg/day p.o		Renal failure, hypertension
Tacrolimus	0.06 mg/kg per day p.o	Usually more effective than cyclosporine and better tolerance	Hypertension, renal insufficiency, gastrointestinal symptoms, infections, and tremor
Mycophenolate mofetil	2–3 g/d p.o	Avoid pregnancy (embryopathy)	Gastrointestinal symptoms, Myelosuppression
Colchicine	0.5 mg/d		Diarrhea, myopathy, neuropathy
Intravenous immunoglobulins	0.4 gr/kg/d × 5 d i.v. in a month × 6 months	Severe disease Difficult venous access	Venous thromboembolism, headache, aseptic meningitis
Rituximab	1 g i.v. given twice within a 2-w interval	Severe disease Maintenance schedule not specified	Infusion-related reaction, infections
Tocilizumab	8 mg per kg i.v./4 w or 162 mg/w s.c	Severe disease Intolerance to other drugs Few cases reported	Liver toxicity, blood cytopenia, infections, intestinal perforation
TNF blockers	Infliximab 3–5 mg/kg/8w i.v	Refractory disease	Screening for latent tuberculosis
Janus kinase inhibitors	Tofacitinib 5 mg/12 h p.o	HZV mandatory Refractory disease	Cancer and venous thromboembolism
Anti-IL5 therapies	Reslizumab 3 mg/kg i.v./4 w Mepolizumab 300 mg s.c./4 w Benralizumab 30 mg s.c. /8 w	Refractory disease	Headaches
Sirolimus	2 mg/d p.o		

*HZV* Herpes zoster vaccine; *TPMT* Thiopurine methyltransferase; *TNF* Tumor necrosis factor; *s.c* subcutaneously; *i.v* intravenously; *w* week

## Data Availability

No datasets were generated or analysed during the current study.
